# Biological Motion Coding in the Brain: Analysis of Visually Driven EEG Functional Networks

**DOI:** 10.1371/journal.pone.0084612

**Published:** 2014-01-14

**Authors:** Daniel Fraiman, Ghislain Saunier, Eduardo F. Martins, Claudia D. Vargas

**Affiliations:** 1 Laboratorio de Investigación en Neurociencia, Departamento de Matemática y Ciencias,Universidad de San Andrés, Buenos Aires, Argentina; 2 CONICET, Buenos Aires, Argentina; 3 Laboratório de Neurobiologia II, Instituto de Biofísica Carlos Chagas Filho, Universidade Federal de Rio de Janeiro, Rio de Janeiro, Brasil; 4 Instituto de Ciências Biológicas, Universidade Federal do Pará, Belem, Brasil; The University of Western Ontario, Canada

## Abstract

Herein, we address the time evolution of brain functional networks computed from electroencephalographic activity driven by visual stimuli. We describe how these functional network signatures change in fast scale when confronted with point-light display stimuli depicting biological motion (BM) as opposed to scrambled motion (SM). Whereas global network measures (average path length, average clustering coefficient, and average betweenness) computed as a function of time did not discriminate between BM and SM, local node properties did. Comparing the network local measures of the BM condition with those of the SM condition, we found higher degree and betweenness values in the left frontal (F7) electrode, as well as a higher clustering coefficient in the right occipital (O2) electrode, for the SM condition. Conversely, for the BM condition, we found higher degree values in central parietal (Pz) electrode and a higher clustering coefficient in the left parietal (P3) electrode. These results are discussed in the context of the brain networks involved in encoding BM versus SM.

## Introduction

It is well known that humans quickly recognize living animals in motion [1]. Detected very early in childhood [2], this capacity has been recently recognized as relevant for social interaction [3]. A successful approach for investigating biological motion introduced by Johannson in the early seventies consists of presenting point lights depicting joint movements in a visual display (point-light display, PLD) [1]. Such stimuli preserve kinematic features while removing distracting information such as color, texture, etc. Notwithstanding, studies have shown that human subjects presented with PLD stimuli maintain the ability to infer key features such as action recognition [4], actor's gender [5], [6] and emotional states [7], [8].

A classical approach for investigating the neural basis of biological motion coding entails measuring the event related potentials (ERP) evoked during the visualization of PLDs depicting human biological locomotion (biological motion, BM) or in a scrambled configuration (scrambled motion, SM). ERPs recorded during the viewing of PLDs portraying human activities reveal a larger negative component for whole-body BM when compared to SM in the 200–350 ms latency range after stimulation onset [3], [9]–[11]. This difference is found mainly in the right occipito-temporal region, reflecting a selective recruitment of the superior temporal sulcus (STS) [3], [9], [10]. A series of brain lesion, transcranial magnetic stimulation (TMS), and fMRI studies have also demonstrated the participation of the parietal lobe [12]–[14] and the premotor cortex [15]–[17] in the recognition of human motion in PLDs. By employing a wider temporal window of analysis, Saunier et al. [11] showed that the difference in ERPs between BM and SM, initially detectable in the right parieto-occipital region, is followed by a similar difference in the fronto-parietal region. Considering that the premotor area, the parietal lobule, and the STS are classically regarded as the cortical core of an action-perception network [18], it was suggested that the biological motion detection process could implicitly map itself onto circuits coding motor vocabularies [11]. Following this ERP study [11], it became evident that several brain regions are involved in discriminating between BM and SM.

In a seminal paper investigating the neural correlates of perceptual coding, Rodriguez et al. [19] showed that the perception of faces, as opposed to meaningless objects, generates a long-distance pattern of synchronization among electrodes. Furthermore, a period of strong desynchronization marks the transition between the moment of perception and an ensuing motor response. These results revealed the existence of a dynamic brain map underlying cognitive task shifts [19]. Such neural assemblies (defined as distributed local networks of neurons transiently linked by reciprocal dynamic connections; for a review, see [20]) can be accessed through the recently developed functional network framework.

Networks of functional interactions have been broadly applied to fMRI data, particularly in experiments employing resting state methodology [21]–[29]. This approach has also been successfully employed with EEG data [30]–[34]. Comparing the resting state EEG functional networks of Alzheimer's disease patients with those of age-matched control subjects [30] revealed differences in the average cluster coefficient and in the average shortest path. Similar results were found when comparing the functional networks of 5 and 7 year-old children [32], indicating a potential use of the neural network approach to delineate groups based on interactions between EEG channels. Less is known about how network parameters are modulated by subtle visual stimuli differences.

In this work, we propose the use of a new fast-scale network methodology to map functional networks extracted from electroencephalographic activity driven by visual motion stimulation. We expected that network graph measures might further our understanding of the cortical networks involved in distinguishing human body locomotion from scrambled nonsense motion on a millisecond scale, shedding new light on the computational coding of cognitive operations in the human brain.

## Materials and Methods

Functional networks derived from time series of EEG data collected from human volunteers are analyzed in detail.

### Participants

A total of sixteen healthy subjects (29.25±6.3 years) with normal or corrected to normal vision and with no known neurological abnormalities participated in this study. The subjects were unaware of the experiments purpose and gave their written informed consent to participate. The study was conducted in accordance with the declaration of Helsinki (1964) and approved by the local ethics committee (Comite de Etica em pesquisa do Hospital Universitario Clementino Fraga Filho, Universidade Federal do Rio de Janeiro, 303.416).

### Stimuli and procedure

Point-light display (PLD) animation was obtained after a session of human walker motion capture (sampling rate of 100 Hz, Elite System, BTS Bioengineering, Italy). The whole-body BM animation depicted ten markers (head, shoulder, elbow, hand, hip, knee and ankle) indicating walker joint coordinates (x, y positions) displayed as white dots against a black background using Presentation software (Neurobehavioral Systems, Inc.). This animation permitted a vivid perception of a walker's movement over a treadmill in the sagittal plane, achieving a complete gait cycle [11] (see video S1).

A single actor's movement repetition was used to create the BM animation and a few SM versions, in which the human locomotion pattern was unrecognizable (see video S2). This non- biological motion control was created permuting the spatial position of the dots, thus destroying the gestalt of the human walker motion while maintaining the biological kinematics of each BM dot. For example the dot in the head was interchanged with that of the knee. A white cross (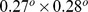) at the center of visual field facilitated gaze fixation and minimized eye movement contamination in the EEG signal.

All animations were shown at 25 frames/sec (to ensure smooth, natural movements) on a 17″ color flat screen [11]. Each participant sat at a comfortable viewing distance from the screen (approximately 70 cm) in a darkened room. The animations were shown in two blocks with a five-minute inter-block interval. Each block consisted of 25 BM and 25 SM stimuli presented randomly. Each stimulus was displayed for 1.3 seconds, followed by an inter-stimulus interval (ISI) of 5 seconds. In each trial, a fixation cross appeared in the last second of the ISI. A total of 100 point light animations were displayed (2 blocks ×2 conditions [BM and SM] ×25 repetitions).

### EEG recordings

EEG activity was recorded using a BrainNet BNT 36 (EMSA) consisting of twenty Ag/AgCl electrodes at the following scalp positions according to the 10–20 system: Fp1, Fp2, F7, F3, Fz, F4, F8, T3, C3, Cz, C4, T4, T5, P3, Pz, P4, T6, O1, Oz, O2. The impedance of each electrode was kept below 5 kΩ. The electrical potential was amplified, bandpass-filtered (0.5–50 Hz), and digitized at a 600 Hz sampling rate, with the mastoid electrodes serving as a reference. The use of mastoids as a reference is widely supported by the biological motion literature [11], [33], [34] and is recommended for set-ups with a small number of electrodes. Artifacts such as oculomotor or muscle activity were rejected offline using a threshold criterion of 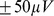.

### Functional network construction and analysis

**Figure 1:**
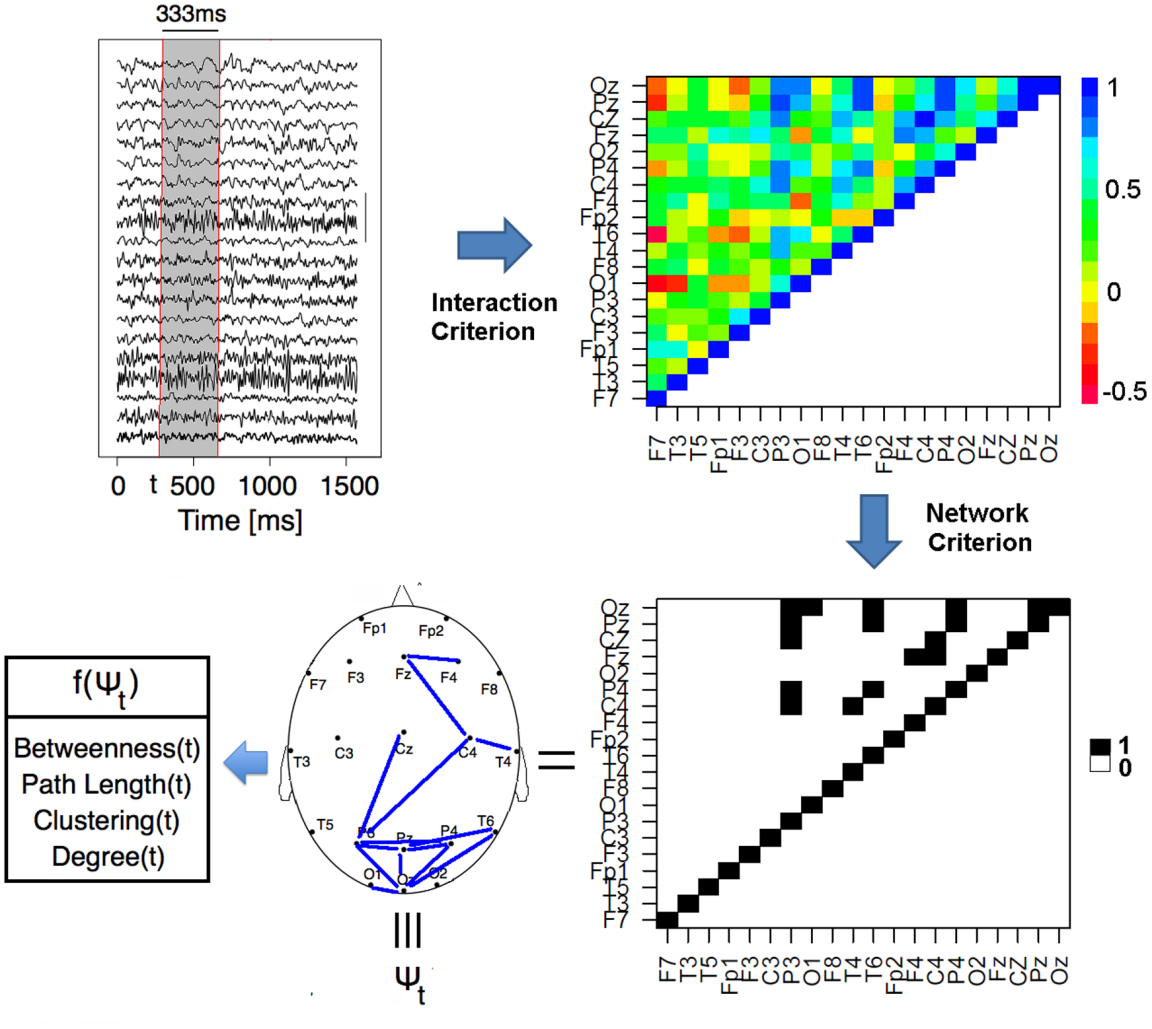
Functional networks construction. Scheme depicting the construction of EEG functional networks and extraction of network properties. In the upper left panel, a moving window of 333-channel EEG data. For each consecutive time windows 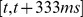 the Spearman (rank) correlation matrix (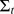) was calculated considering the functional correlations of 20×19/2 pairs of electrodes (upper right panel). A binary matrix (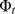, lower right panel), converted from the continuous matrix by means of a network criterion (see text), was employed to construct the EEG functional networks and to extract the network properties betweeness, path length, clustering and degree of each node (lower left panel).

After filtering the 20-channel EEG data, a comparison of BM vs. SM power spectrum was done for each frequency in the 0–50 Hz range. Statistical significance was assessed by employing the Wilcoxon test. No difference was observed at level of significance of 0.05. Thus, without any a priori reason to further explore functional networks at different frequency bands, the functional correlation analysis was done with bandpass (0.5–50 Hz) filtered signals. Functional networks evolving over time (Fig. 1) were constructed by considering a moving window of 333 ms. Consecutive time windows were shifted by 43.3 ms. The Spearman (rank) correlation matrix, 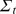, was calculated for each temporal window [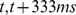]. This matrix contained the 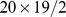 pairs of electrode functional correlations (or interactions). The Spearman correlation is defined as the Pearson correlation between the ranked signals. Given two time series 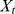, and 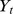 with 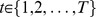, a rank time series for 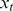 and 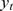 is constructed (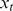 and 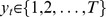) and the Pearson correlation between the last two series is computed as follows:
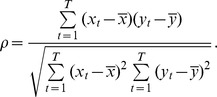
It is important to note that only correlations between zero-lag time series were considered. As shown in Fig. S1, the rank correlation decays from the maximum value, which occurs at lag zero, to values near zero for lags on the order of 50 ms, where fluctuations appears. The absence of maximums for non-zero-lag led us to choose a zero-lag correlation criterion.

Once the correlation matrix was computed, we applied a criterion for converting the continuous matrix to a binary matrix, 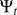, thus defining a network (Fig. 1). Changing this criterion resulted in distinct functional networks. As shown in Fig. S2 A-B, the EEG functional networks varied in time whether they were constructed with a fixed number of links (or fixed average degree) or a fixed correlation threshold criterion. Furthermore, the two criteria resulted in different networks because of large fluctuations in the average correlation (Fig. S2C). EEG data contains periods of large (global) levels of synchronization between electrodes and other periods without this synchronization [31].

This type of brain dynamics behavior has also been reported [35] in resting state fMRI. This synchronization effect results in networks that vary greatly in the number of links when using the fixed correlation threshold criterion, while the fixed number of links criterion significantly reduces network variability. Therefore, all EEG functional networks were constructed using the fixed number of links criterion.

### Extracting functional network measures

Once the network was constructed, summary measures, or functions of the adjacency matrix, 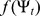, were computed. More specifically, we selected properties that characterized each node (local network measures) and properties that characterize the networks as a whole (global network measures). The average shortest path, or average path length, is one of the global measures we studied in detail. The ability of a network of N nodes to propagate information depends primarily on the separation between nodes. The average separation between two nodes in a graph is given by the average shortest path (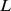) defined as the average of geodesic lengths over all pairs of nodes:
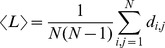
where the shortest path (or minimum links of separation) between nodes 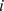 and 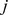 is represented by the variable 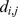. Brain functional networks can also be characterized by the level of segregation [25], [27], [36], measured as the number of functional clusters (i.e. a group of nodes) recruited during a particular task. A local measure of segregation is given by the clustering coefficient of a given node 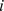, 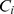. This measure is defined as the number of connections between all neighbors of node 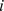, 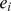, divided by the total number of possible links between them equal to 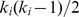, where 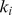 is the degree (number of links) of node 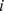.
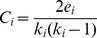
In this case the global measure is the average clustering coefficient.
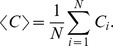
As discussed in [27] another important brain network parameter is the “central role” of a particular node. Different measures of node centrality are described, with degree being the best known. In theory, a brain region with large degree (interacting with many other regions) is a region that facilitates functional integration. However, a node can have a low degree but still be very important in integrating two segregated regions, depending on the amount of information that passes through it. This property describes another measure of node centrality, the betweenness coefficient. For a node 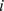, this coefficient is defined as the fraction of all shortest paths in the network that pass through this node:
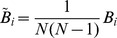
where
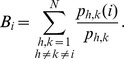


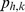
 is the number of shortest paths between nodes 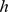 and 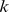, and 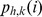 is the number of shortest paths between 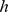 and 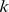 that pass through 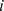 (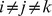). To ensure the best representation of the functional network, we directly studied 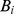, which alludes to the amount of flow passing through node 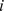. The average betweenness is defined as follows:
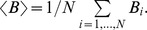


### Statistical analysis

Global and local network parameters extracted trial by trial from each functional network analysis window and for each condition (BM or SM) were compared statistically using the Wilcoxon (rank) test. Significant differences between BM and SM should meet two criteria: first, the p-values should be less than 0.05/20 (Bonferroni correction); second, significant differences should be verified in at least three networks with different numbers of links.

## Results

**Figure 2:**
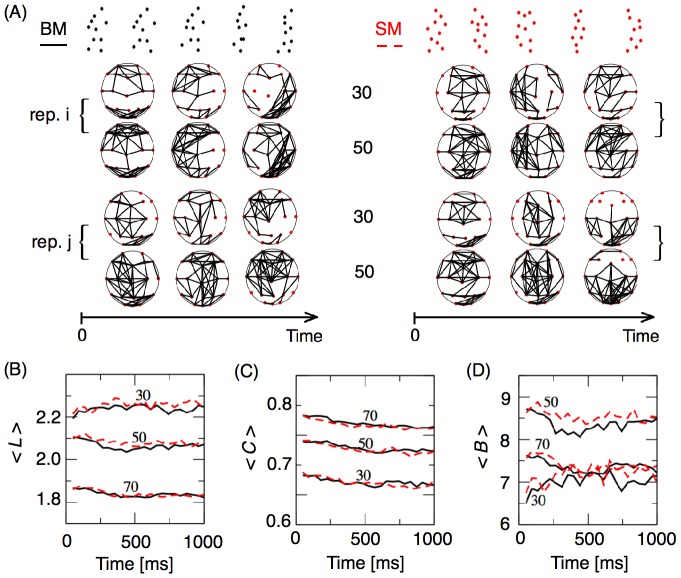
Comparing global properties of biological motion (BM, black lines) and scrambled motion (SM, red dotted lines). (A) Functional networks evolving in time built from data gathered in two repetitions (i and j) of each condition (BM and SM) by employing the 30 and the 50 highest correlated links. Functional network properties: Average (B) path length, (C) clustering coefficient, and (D) betweenness depicted in function of time for networks built from the highest 30, 50, and 70 links in BM and SM conditions.

Fig. 2A illustrates BM and SM functional networks constructed with 30 or 50 links, evolving over time for two different repetitions. These functional networks were characterized by large variability, regardless of the condition, repetition, number of links, or moment in time.

### Global network measures

Global network measures extracted from BM and SM conditions were compared as a function of time (Fig. 2 panels B-D). The average path length, 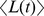, a global property that reflects the average distance between nodes, was insensitive to any difference between BM and SM regardless of the link density (Fig. 2 B). Moreover, the average path length of networks with 30, 50, or 70 links showed no temporal modulation. For example, BM or SM functional networks with 70 (30) links had an 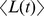 that fluctuated around 1.85 (2.25). Fig. 2C shows the clustering time evolution of BM and SM functional networks. This global network measure tended to decrease slightly over time, but it was not sensitive to the difference between BM and SM. The average betweenness coefficient, 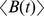, also did not differentiate BM and SM (Fig. 2D). Thus, standard network (global) measures cannot discriminate between BM and SM. Interestingly, global parameters extracted from stimuli-driven functional networks are clearly distinct from those calculated from random networks (see Fig. S3).

### Local network measures

**Figure 3:**
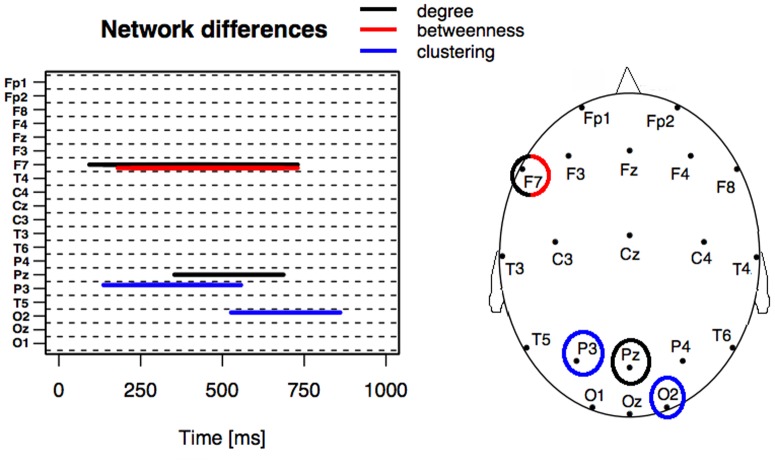
Comparing local properties of functional networks extracted from biological motion (BM) and scrambled motion (SM) conditions for each electrode as a function of time. Color lines represent local network properties (degree, betweeness and clustering coefficient) for which significance level between BM and SM was attained (p-value <0.05/20).

Differences between BM and SM in local network measures were found for degree, betweenness, and clustering coefficient. Fig. 3 illustrates the moments in time when these differences (corrected for multiple comparisons) were identified. A topological schema indicating local network differences between BM and SM for each of the tested electrodes is shown in the right panel. For instance, differences in degree and betweenness were detected between BM and SM in electrode F7 during a large time period from 100 ms to 750 ms. In electrode P3, the clustering coefficient differed between BM and SM from 150 ms to 550 ms. A difference in the clustering coefficient between BM and SM was also found in electrode O2 from 500 ms to 850 ms. Finally, a difference in degree between BM and SM was detected in electrode Pz from 350 ms to 700 ms.

**Figure 4:**
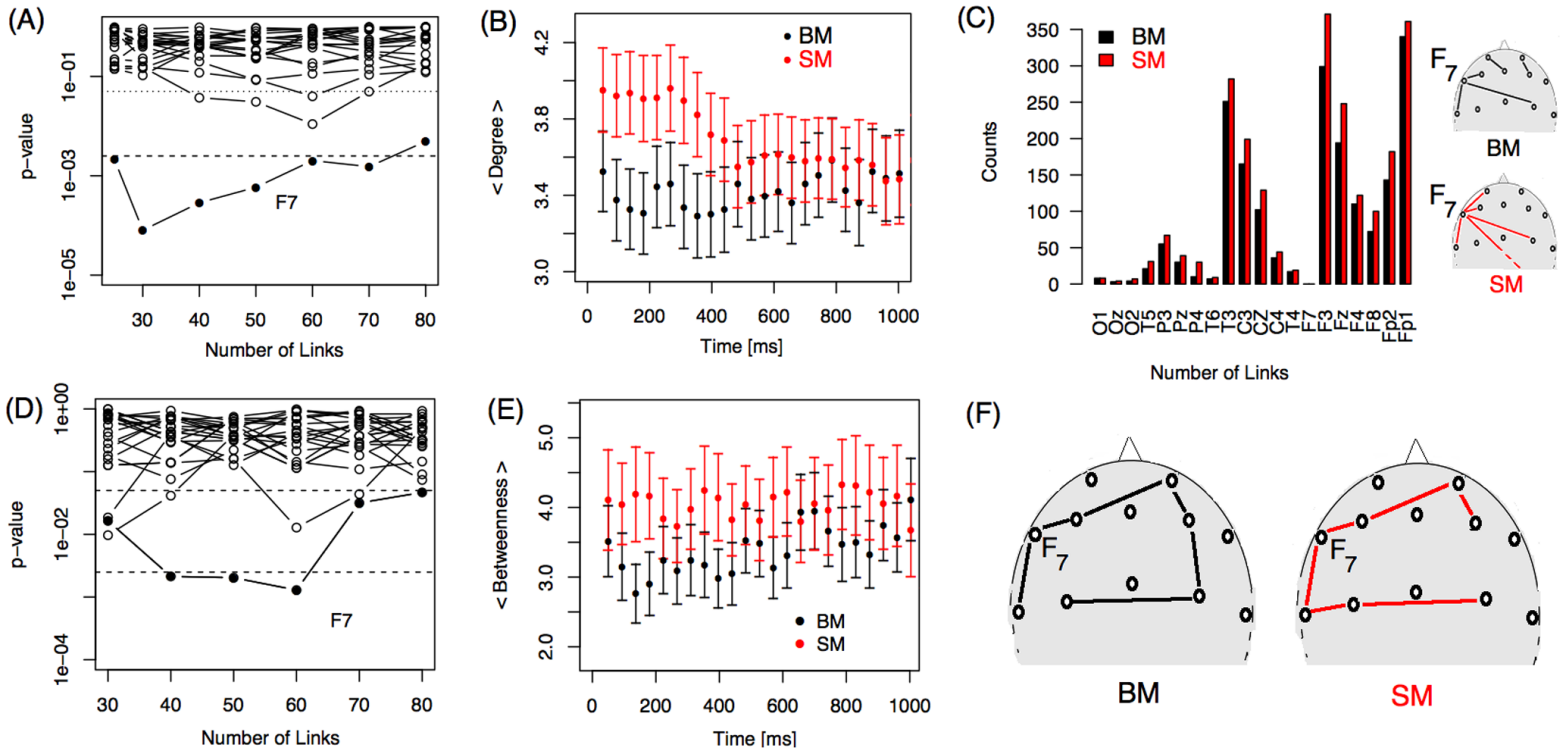
Comparing local properties of biological motion (BM) and scrambled motion (SM) in electrode F7. P-values from the Wilcoxon test for (A) degree and (D) betweenness for BM and SM at different numbers of links. Each line corresponds to one electrode studied during a time widow starting at (A) 310 ms or (D) 137 ms. Average (B) degree and (E) betweenness for electrode F7 is plotted as a function of time for both conditions, for networks built with 50 links, with a 95% confidence interval. (C) Histogram depicting the first neighbors of F7 with larger degree values for SM compared to BM, as illustrated in the pictograms. (F) Pictorial representation of a scenario explaining the differences in betweenness between conditions.

Fig. 4 displays the p-values obtained from a Wilcoxon test for degree and betweenness coefficients for each electrode and for seven link densities. A significant difference in degree (p-value <0.05/20) between BM and SM was identified for electrode F7 for several densities of links (Fig. 4A). The time evolution of the F7 average degree (Fig. 4B) reveals that this parameter was larger for SM within the first 750 ms (each graph point represents a time window of 333 ms; see Materials and Methods). We then looked for new links appearing for electrode F7 in the SM condition that could contribute to a higher degree value by determining which nodes (electrodes) were connected with electrode F7 by a single link (first neighbors of F7). Fig. 4C summarizes the number of times each node was counted as a first neighbor of F7 in both motion conditions from a total of 550 networks. When compared with the BM condition, F7 is more connected with almost all other electrodes in the SM condition (except electrode O1, which connects to F7 exactly the same number of times in both conditions). Thus, no specific new link appears in, or codes for, the SM condition. A larger betweenness coefficient was also found for this electrode in the SM condition (Figs. 4D and E). Two simultaneous effects might contribute to this increase in the betweenness coefficient of F7. On one hand, an increased degree favors an increase in betweenness because more links pass through the electrode. On the other hand, a network reorganization maintaining a fixed degree, such as the one represented in Fig. 4F, could occur. In this scheme, we show the effect of changing one link over F7 betweenness. This gives electrode F7 a larger centrality value in the SM condition; that is, more paths pass through F7 in SM.

**Figure 5:**
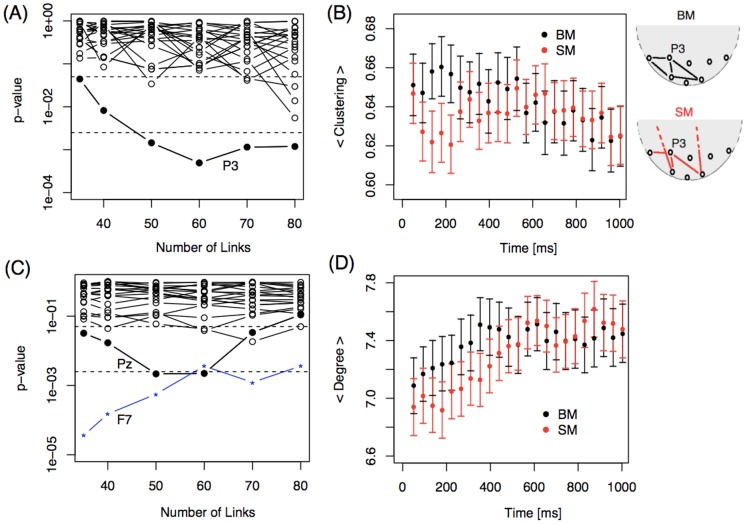
Comparing local properties of biological motion (BM) and scrambled motion (SM) in electrodes P3 and Pz. P-values of the Wilcoxon test for (A) clustering and (C) degree for BM and SM at different densities of links. Each line corresponds to one electrode studied during a time window starting at (A) 137 ms or (C) 353 ms. Average (B) clustering coefficient for electrode P3 and (D) degree for electrode Pz plotted as a function of time for both conditions in networks built with 50 links with a 95% confidence interval. Upper right: Pictograms illustrating the higher clustering effect of BM compared to SM.

As depicted in Fig. 5, the clustering coefficient of electrode P3 differs between BM and SM. This coefficient was larger in the BM condition (Fig. 5, panels A and B), indicating that its neighbors are more interconnected in BM than in SM from 150 ms to 550 ms. This suggests that P3 neighbors are segregated from the whole network in the BM condition. Fig. 5C illustrates this process. Electrode Pz exhibited a difference in degree between BM and SM later in time (Fig. 5D), again with higher values in the BM condition.

Electrode O2 was also involved in the process of differentiating between BM and SM, with a clustering coefficient larger in SM from 500 ms to 800 ms (data not shown).

Finally, no differences in local network properties were found by comparing functional networks computed from the fixation cross period preceding BM and SM visual stimulation, indicating that the above-described results are specific to BM and SM encoding in the brain.

## Discussion

In this work, we have asked whether graph measures extracted from EEG functional networks evolving over time differentiate point-light displays depicting human body locomotion and those depicting scrambled motion. We have shown that the BM and SM functional networks behave similarly in regard to global network properties but resolve from each other in specific network nodes when local properties are considered.

A concern that arises when working with high-density acquisition systems is the effect of volume conduction on EEG functional networks. As mentioned, part of network construction consists of defining a synchronization measure between nodes. Like other measures of synchronization [19], [37], [38], the one used here does not omit conduction effects. For instance, Peraza et al. [39] recently showed that coherence, phase coherence, and phase lag index are all affected by volume conduction. Notwithstanding, the functional network approach allows us to make inferences about EEG brain activity interactions under different conditions [31], [33], [34]. In the same vein, synchronization measures have been successfully used to map interactions associated with face versus nonsense visual stimuli [19]. Likewise, the devised experimental paradigm used herein was such that the stimuli were identical except for the invoked shape of a walking human in the BM condition. Hence, volume conduction effects should have affected both conditions comparably. As such, we assume that the observed differences between BM and SM are not a consequence of volume conduction.

The main result of the present study involves functional network local properties. Specifically, the local properties computed from electrodes F7, P3, Pz, and O2 were sensitive to subtle differences between the two visual stimuli, suggesting that whole-body locomotion and non-sense visual stimuli draw on distinct local networks. Interestingly, differences between BM and SM were first detected in electrode F7, only later appearing in parietal and occipital electrodes. These results are discussed in detail below.

In electrode F7 (corresponding roughly to the scalp region over Brodmann's areas 45 and 47 [40]), the first scalp site to show a difference between the BM and SM conditions over time, both the degree and the betweenness coefficient were larger in SM compared to BM. This effect lasted for several hundreds of milliseconds. The increase in degree found in electrode F7 is indicative of a higher number of regions interacting with this electrode in the SM condition. In other words, more electrodes associated with F7 became linked with each other during the coding of a non-sense, scrambled stimulus compared to that of a readily recognizable [1] human biological motion stimulus. The role of the left frontal electrode F7 in discriminating BM vs SM was also revealed by use of repetitive TMS. This technique permits to temporarily block the brain activity underlying the stimulated region [41]. Repetitive transcranial magnetic stimulation (rTMS, theta bursts) applied over the scalp region corresponding to the left ventral premotor cortex (PMC, Brodmanns' area 45) reduce sensitivity to biological motion perception, possibly by compromising access to and/or read-out of the perceiver's own motor representations [17]. In particular, temporarily silencing the PMC's neural activity by means of rTMS increases the participant's tendency to indicate that biological motion was present when it was not [17]. Likewise, the local degree and betweenness coefficient of electrode F7 were herein able to tell apart familiar, clearly coded human locomotion from an unrecognizable (and possibly more demanding) visual context. These results support the hypothesis that the inferior frontal lobule, which contains a representation of movement kinematics [42], [43], is a core node in PLD action recognition. Our results also imply that non-sense, unrecognizable visual stimuli involves a more extended brain network. It is possible that the processing of familiar stimuli, such as whole-body BM, recruits committed representations in the brain, whereas the coding of nonsense stimuli involves a less constrained network. Interestingly, a delayed higher clustering coefficient for SM was found in electrode O2, suggesting a further recruitment of the occipital lobe in SM stimulus processing.

In the BM condition, higher clustering and degree values were found in the left (P3) and central (Pz) parietal electrodes, respectively, showing that this sub-network is more strongly recruited during BM as opposed to SM processing. These results confirm the participation of the parietal lobe in the coding of biological PLD [14], [44], [45]. Likewise, the parietal cortex has been shown to contribute to the read-out of motor vocabularies triggered by action observation [46]–[49]. Taken together, our results show that local functional network properties draw on a parietofrontal network, that has been repeatedly demonstrated to be an essential component of biological motion coding.

Early brain imaging data indirectly favors the conjecture that attention is involved in biological motion processing, as regions consistently enrolled in attentional coding such as the anterior portion of the intraparietal sulci, the inferior and superior parietal lobule, the amygdala and cerebellum have also been shown to take part in the processing of point-light displays [12]–[14], [45], [50]–[54]. Indeed, even if the perception of biological motion depicting human locomotion appears effortless, such process could require attentional load [55]. In a careful ERP and source localization study, Jokisch et al. [3] found that sources for the N170 component generated by the contrast between BM and SM were located in the posterior cingulate cortex. As this area has been suggested to mediate the anticipatory allocation of spatial attention [56], Jokish et al. [3] argued that its activation could possibly reflect high-level spread attention subserving neural processing leading to the BM global percept. Applied to the present study, the higher clustering and degree values found in P3 and Pz for BM as opposed from SM condition could thus result at least in part from distinct attentional demands. Further work might help disentangle attentional x biological motion coding in the brain.

Levels of synchronization between areas of interest upon the presentation of biological motion in different visual contexts (point-light vs. real human motion) have been investigated using the phase-lag index (PLI) [33]. The results showed that functional connectivity is greater within the supplementary motor and left temporal areas during the unfamiliar display (i.e. PLD) compared with the familiar display (i.e. video maintenance condition). In the same vein, the authors of [34] showed that the synchronization likelihood, a general measure of linear and non-linear correlations between EEG signals [37], [57], discriminates between brain activity networks evoked by the observation of biological motion presented in point-light versus those presented in video displays. Unlike [34], our study used the same visual context (i.e. PLD) and only manipulated the biological motion information. Our results thus differ from these studies [33], [34] by establishing cognitive correlates of fine topology network parameters calculated from zero-lag functional interactions between electrodes. We chose a data-driven approach where all the information available for each electrode over time was employed to construct graphs of interactions, allowing for a fine-grained comparison between conditions without any *a priori* regions of interest.

In conclusion, the use of a new fast-scale network methodology was herein proposed for the mapping of functional networks extracted from electroencephalographic activity driven by visual stimulation. The local network graph parameters of degree, betweenness, and clustering allowed us to distinguish between biological and scrambled motion conditions in precise moments in time and for specific node points. Higher clustering and degree values herein found for respectively P3 and Pz electrodes indicate that these nodes may be crucial in orchestrating a pattern of activity through time allowing sorting out BM from SM. Accordingly, the parietal cortex has been shown to encode body kinematics [58]. As for F7, higher betweeness and degree values found very early in BM x SM processing confirms that this left frontal region plays a central role in disentangling biological from non sense motion. Whereas F7 electrode provides motor kinematic representations of the action, O2 would appear as its visual counterpart. Indeed, the lateral occipital region was described as encoding the visual kinematic representation of observed actions [43]. Thus, subtle differences herein found between BM and SM concern cortical regions (inferior frontal lobule, parietal cortex and lateral occipital cortex) involved in the analysis of the kinematic features of the action. In addition, differences in latency that occur between conditions ([100–750 ms] for F7 and [500–850 ms] for O2 electrode) suggest a possible top-down influence from frontal to occipital regions. Taken together these results are consistent with the proposal of a wide complex neural network within the sensorimotor system devoted to the processing of biological motion [11]. These results add on to the understanding of cortical networks involved in the coding of biological motion. Thus, the functional network approach is a suitable method for studying brain function on the time scale of cognitive processing and it allows for a new level of understanding of the complex phenomena associated with brain function.

## Supporting Information

Figure S1
**Defining criteria to construct functional networks: lag correlations.** Spearman correlation as a function of the lag. Three pairs of electrodes, C3-P4 (left panel), Fp2-T4 (middle panel) and F7-F3 (right panel) are shown for 6 different subjects. Each color curve corresponds to the average (over 25 repetitions) for one subject in the BM condition. The correlation between the time series 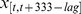 and 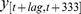 was computed for 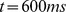. The behavior showed here is verified for all pairs of electrodes. Interchanging *x* by *y* in the correlation formula we obtain similar results.(TIF)

Figure S2
**Defining criteria to construct functional networks: correlation threshold vs. fixed number of links.** (A) EEG functional networks were constructed employing two different criteria: fixed number of links (upper row) and fixed correlation threshold (bottom row). (B) Empirical distribution function 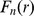 of the correlation, *r*, between two electrode signals. Each color curve corresponds to a different moment in time, and contains the 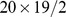 pairs of correlations between the twenty electrode signals. (C) Average correlation, 
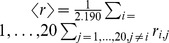
, as a function of time. Data corresponding to one subject observing biological motion.(TIF)

Figure S3
**Comparison EEG functional networks with Erdös-Rényi networks of the same number nodes and links.** (A) Average path length, (B) average clustering coefficient, and (C) average betweenness as a function of the number of links. Brain EEG functional networks present a small world structure (panel A and B), i.e. large value of the ratio 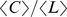. The average betweeness coefficient (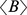) of EEG networks (panel C) shows a non monotonic relationship with the number of links. Random networks, contrary to functional brain networks, satisfy a monotonic decreasing relationship.(TIF)

Video S1 (MOV)

Video S2 (MOV)
