# Supplementary figures and images for: Biological Motion Coding in the Brain: Analysis of Visually Driven EEG Functional Networks
Source: PLoS One. 2014 Jan 14;9(1):e84612. doi: 10.1371/journal.pone.0084612 (PMC3891803; doi:10.1371/journal.pone.0084612)

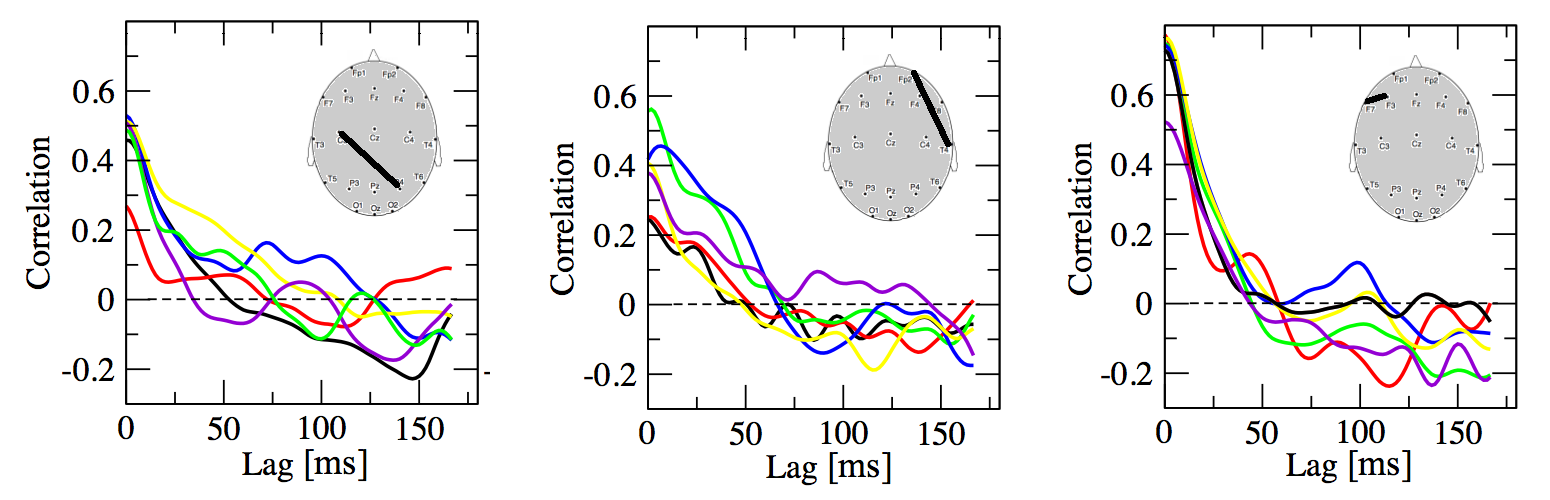

Supplement: Figure S1 — Defining criteria to construct functional networks: lag correlations. Spearman correlation as a function of the lag. Three pairs of electrodes, C3-P4 (left panel), Fp2-T4 (middle panel) and F7-F3 (right panel) are shown for 6 different subjects. Each color curve corresponds to the average (over 25 repetitions) for one subject in the BM condition. The correlation between the time series and was computed for . The behavior showed here is verified for all pairs of electrodes. Interchanging x by y in the correlation formula we obtain similar results. (TIF) [file pone.0084612.s001.tif]

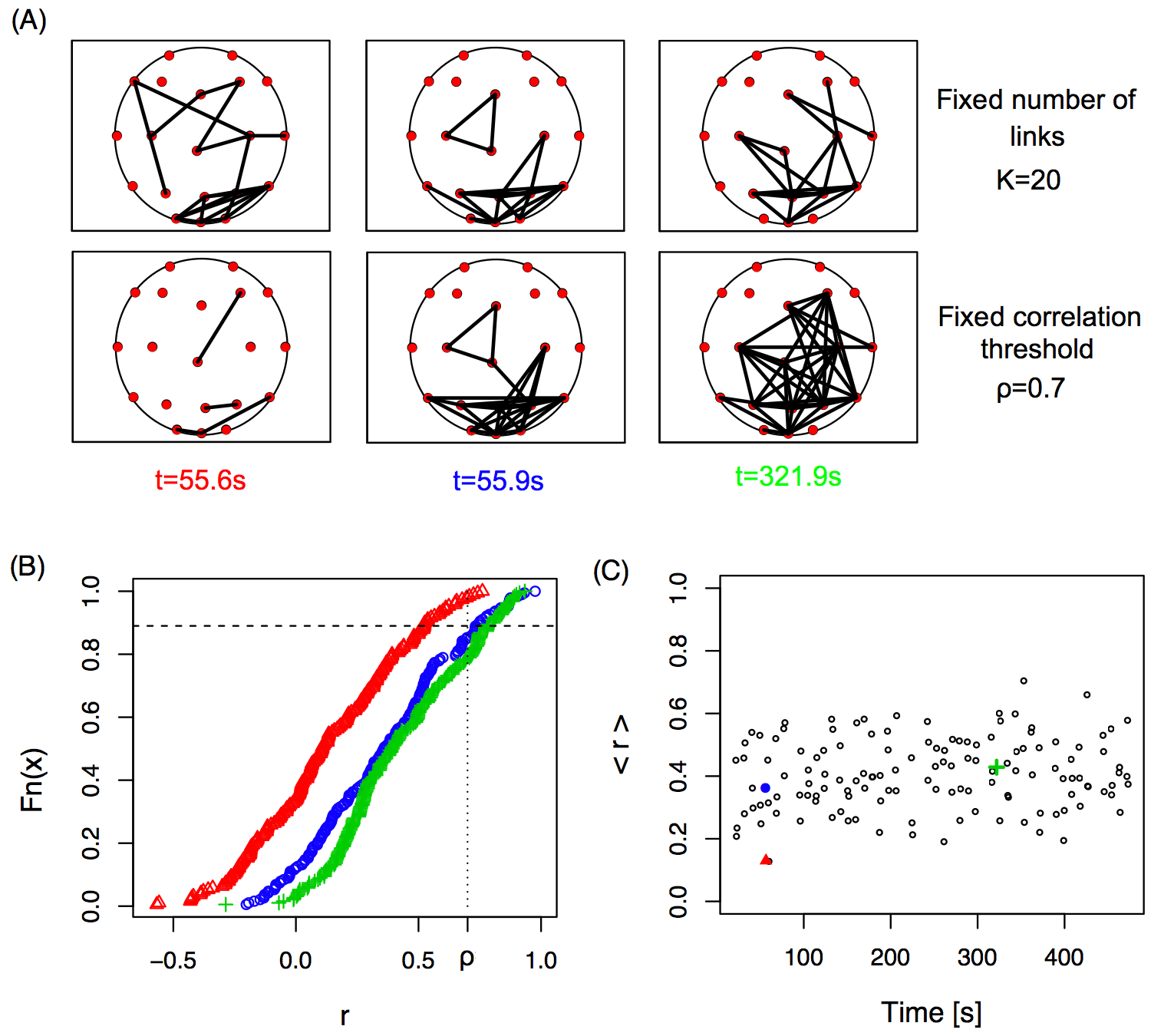

Supplement: Figure S2 — Defining criteria to construct functional networks: correlation threshold vs. fixed number of links. (A) EEG functional networks were constructed employing two different criteria: fixed number of links (upper row) and fixed correlation threshold (bottom row). (B) Empirical distribution function of the correlation, r, between two electrode signals. Each color curve corresponds to a different moment in time, and contains the pairs of correlations between the twenty electrode signals. (C) Average correlation, , as a function of time. Data corresponding to one subject observing biological motion. (TIF) [file pone.0084612.s002.tif]

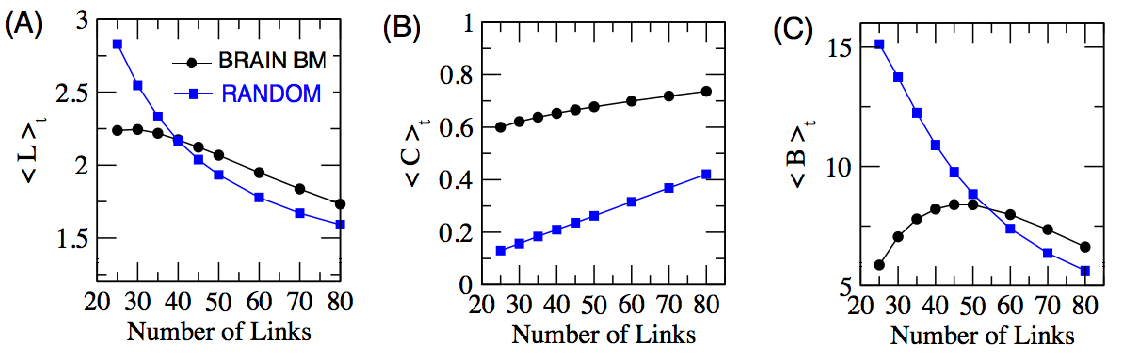

Supplement: Figure S3 — Comparison EEG functional networks with Erdös-Rényi networks of the same number nodes and links. (A) Average path length, (B) average clustering coefficient, and (C) average betweenness as a function of the number of links. Brain EEG functional networks present a small world structure (panel A and B), i.e. large value of the ratio . The average betweeness coefficient () of EEG networks (panel C) shows a non monotonic relationship with the number of links. Random networks, contrary to functional brain networks, satisfy a monotonic decreasing relationship. (TIF) [file pone.0084612.s003.tif]
